# On the Expressiveness of Büchi Arithmetic

**DOI:** 10.1007/978-3-030-71995-1_16

**Published:** 2021-03-23

**Authors:** Christoph Haase, Jakub Różycki

**Affiliations:** 8 University of Oxford, Oxford, UK; 9 Sorbonne Université - LIP6, Paris, France; 10 Department of Computer Science, University of Oxford, Oxford, UK; 11 Institute of Mathematics, University of Warsaw, Warsaw, Poland

**Keywords:** logical theories, logical definability, quantifier elimination, automatic structures, regular languages

## Abstract

We show that the existential fragment of Büchi arithmetic is strictly less expressive than full Büchi arithmetic of any base, and moreover establish that its $\varSigma _2$-fragment is already expressively complete. Furthermore, we show that regular languages of polynomial growth are definable in the existential fragment of Büchi arithmetic.

## 1 Introduction

This paper studies the expressive power of Büchi arithmetic, an extension of Presburger arithmetic, the first-order theory of the structure $\langle \mathbb{N}, 0, 1, + \rangle$. Büchi arithmetic additionally allows for expressing restricted divisibility properties while retaining decidability. Given an integer $p \geq 2$, *Büchi arithmetic of base p* is the first-order theory of the structure $\langle \mathbb{N}, 0, 1, +, V_p \rangle$, where $V_p$ is a binary predicate such that $V_p(a, b)$ holds if and only if $a$ is the largest power of $p$ dividing $b$ without remainder, i.e., $a = p^k$, $a \mid b$ and $p \cdot a \nmid b$.

Presburger arithmetic admits quantifier-elimination in the extended structure $\langle \mathbb{N}, 0, 1, +, \{c|\cdot\}_{c>1} \rangle$ additionally consisting of unary divisibility predicates $c|\cdot$ for every $c > 1$ [10]. It follows that the existential fragment of Presburger arithmetic is expressively complete, since any predicate $c|\cdot$ can be expressed using an additional existentially quantified variable. We study the analogous question for Büchi arithmetic and show, as the main result of this paper, that its existential fragment is, in any base, strictly less expressive than full Büchi arithmetic. Notably, this result implies that there does not exist a quantifier-elimination result *à la* Presburger for Büchi arithmetic, i.e., any extension of Büchi arithmetic with additional predicates definable in existential Büchi arithmetic does not admit quantifier elimination.

A central result about Büchi arithmetic is that it is an automatic structure: a set $M \subseteq \mathbb{N}^n$ is definable in Büchi arithmetic of base $p$ if and only if $M$ is recognizable by a finite-state automaton under a base $p$ encoding of the natural

⋆ Parts of this research were carried out while the first author was affiliated with the Department of Computer Science, University College London, UK.

numbers. Equivalently, $M$ is *p-regular.* This result was first stated by Büchi [4], albeit in an incorrect form, and later correctly stated and proved by Bruyère [2], see also [3]. Villemaire showed that the $\Sigma_3$-fragment of Büchi arithmetic is expressively complete [13, Cor. 2.4]. He established this result by showing how to construct a $\Sigma_3$-formula defining the language of a given finite-state automaton. We observe that Villemaire's construction can actually be improved to a $\Sigma_2$-formula and thus obtain a full characterization of the expressive power of Büchi arithmetic in terms of the number of quantifier alternations.

Our approach to separating the expressiveness of existential Büchi arithmetic from full Büchi arithmetic in base $p$ is based on a counting argument. Given a set $M \subseteq \mathbb{N}$, define the counting function $d_M(n) := \#(M \cap \{p^{n-1}, \ldots, p^n - 1\})$ which counts the numbers of bit-length $n$ in base $p$ in $M$. If $M$ is definable in existential Büchi arithmetic of base $p$, we show that $d_M$ is either $O(n^c)$ for some $c \geq 0$, or at least $c \cdot p^n$ for some constant $c > 0$ and infinitely many $n \in \mathbb{N}$. Since, for instance, for $M_p \subseteq \mathbb{N}$ defined as the set of numbers with $p$-ary expansion in the regular language $\{10, 01\}^*$, we have $d_{M_p}(n) = \Theta(2^{n/2})$, and hence $M_p$ is not definable in existential Büchi arithmetic of base $p$. However, $M_p$ being $p$-regular implies that $M_p$ is definable by a $\Sigma_2$-formula of Büchi arithmetic of base $p$.

We also show that existential Büchi arithmetic defines all regular languages of polynomial density, encoded as sets of integers. Given a language $L \subseteq \Sigma^*$, let the counting function $d_L \colon \mathbb{N} \to \mathbb{N}$ be such that $d_L(n) := \#(L \cap \Sigma^n)$. Szilard et al. [11] say that $L$ has *polynomial density* whenever $d_L(n)$ is $O(n^c)$ for some non-negative integer $c$. If moreover $L$ is regular then Szilard et al. show that $L$ is represented as a finite union of regular expressions of the form $v_0 w_1^* v_1 \cdots w_k^* v_k$ such that $0 \leq k \leq c + 1$, $v_0, w_1, v_1, \ldots, v_k, w_k \in \Sigma^*$ [11, Thm. 3]. We show that existential Büchi arithmetic defines any language represented by a regular expression $v_0 w_1^* v_1 \cdots w_k^* v_k$, which implies that existential Büchi arithmetic defines all regular languages of polynomial density.

## 2 Preliminaries

Given $\boldsymbol{v} = (v_1, \ldots, v_d) \in \mathbb{Z}^d$, we denote by $\|\boldsymbol{v}\|_\infty$ the maximum norm of $\boldsymbol{v}$, i.e., $\|\boldsymbol{v}\|_\infty = \max\{|v_1|, \ldots, |v_d|\}$. For a matrix $\mathbf{A} \in \mathbb{Z}^{m \times d}$ with entries $a_{i,j}$, $1 \leq i \leq m$, $1 \leq j \leq d$, we denote by $\|\mathbf{A}\|_{1,\infty}$ the one-infinity norm of $\mathbf{A}$, i.e., $\|\mathbf{A}\|_{1,\infty} = \max\{|a_{i,1}| + \cdots + |a_{i,d}| : 1 \leq i \leq m\}$.

Let $\Sigma$ be an alphabet and $w \in \Sigma^*$, we denote by $|w|$ the length of $w$. Given a set $U \subseteq \mathbb{N}$, we denote by $w^U := \{w^u : u \in U\}$. Thus, for example, $w^* = w^{\mathbb{N}}$.

For an integer $p \geq 2$, let $\Sigma_p := \{0, \ldots, p-1\}$. We view words over $\Sigma_p$ as numbers encoded in $p$-ary most-significant bit first encoding. Tuples of numbers of dimension $n$ can be encoded as words over the alphabet $\Sigma_p^n$. For $w = \boldsymbol{v}_m \cdots \boldsymbol{v}_0 \in (\Sigma_p^n)^{m+1}$, we denote by $[\![w]\!]_p \in \mathbb{N}^n$ the $n$-tuple

$$[\![w]\!]_p := \sum_{i=0}^{m} \boldsymbol{v}_i \cdot p^i \,.$$

We furthermore define $[\![\varepsilon]\!]_p := 0$. Note that $[\![\cdot]\!]_p$ is not injective since, e.g., 01 and 001 both encode the number one. Given $L \subseteq (\Sigma_p^n)^*$, we define

$$[\![L]\!]_p := \{[\![w]\!]_p : w \in L\} \subseteq \mathbb{N}^n\,.$$

*Automata.* A *deterministic automaton* is a tuple $A = (Q, \Sigma, \delta, q_0, F)$, where

- $Q$ is a set of *states*,
- $\Sigma$ is a finite alphabet,
- $\delta\colon Q \times \Sigma \to Q \cup \{\bot\}$, where $\bot \notin Q$, is the *transition function*,
- $q_0 \in Q$ is the *initial state*, and
- $F \subseteq Q$ is the set of *final states*.

For states $q, r \in Q$ and $u \in \Sigma$, we write $q \xrightarrow{u} r$ if $\delta(q, u) = r$, and extend $\rightarrow$ inductively to words by stipulating, for $w \in \Sigma^*$ and $u \in \Sigma$, that $q \xrightarrow{w \cdot u} r$ if there is $s \in Q$ such that $q \xrightarrow{w} s \xrightarrow{u} r$. The *language of* $A$ is defined as $L(A) = \{w \in \Sigma^* : q_0 \xrightarrow{w} q_f, q_f \in F\}$.

Note that *a priori* we allow automata to have infinitely many states and to have partially defined transition functions (due to the presence of $\bot$ in the co-domain of $\delta$). If $Q$ is finite then we call $A$ a *deterministic finite automaton (DFA)*, and if in addition $\Sigma = \Sigma_p^n$ for some $p \geq 2$ and $n \geq 1$ then $A$ is called a *p-automaton*. Throughout this paper, we assume, without loss of generality, that all states of a DFA are live, i.e., every state is reachable from the initial state and can reach an accepting state.

*Arithmetic theories.* As stated in the introduction, Presburger arithmetic is the first-order theory of the structure $\langle \mathbb{N}, 0, 1, + \rangle$, and Büchi arithmetic of base $p$ the first-order theory of the extended structure $\langle \mathbb{N}, 0, 1, +, V_p \rangle$. We write atomic formulas of Presburger arithmetic as $\boldsymbol{a} \cdot \boldsymbol{x} = c$, where $\boldsymbol{a} = (a_1, \ldots, a_d)^\intercal$ with $a_i \in \mathbb{Z}$, $c \in \mathbb{Z}$, and $\boldsymbol{x} = (x_1, \ldots, x_d)$ is a vector of unknowns. In Büchi arithmetic we additionally have atomic formulas $V_p(x, y)$ for the unknowns $x$ and $y$. For technical convenience, we assert that $V_p(x, 0)$ never holds.[3] We write $\Phi(x)$ or $\Phi(\boldsymbol{x})$ to indicate that $x$ or a vector of unknowns $\boldsymbol{x}$ occurs free in $\Phi$. If there are further free variables in $\Phi$, we assume them to be implicitly existentially quantified.

We may without loss of generality assume that no negation symbol occurs in a formula of Büchi arithmetic. First, we have $\neg(\boldsymbol{a} \cdot \boldsymbol{x} = c) \equiv \boldsymbol{a} \cdot \boldsymbol{x} \leq c - 1 \vee \boldsymbol{a} \cdot \boldsymbol{x} \geq c + 1$, and the order relation $\leq$ can easily be expressed by introducing an additionally existentially quantified variable. Moreover, we have

$$\neg V_p(x, y) \equiv y = 0 \vee \exists z\colon V_p(z, y) \wedge \neg(x = z)\,.$$

Finally, $P_p(x) := V_p(x, x)$ denotes the macro asserting that $x$ is a power of $p$.

Given a formula $\Phi(\boldsymbol{x})$ of Büchi arithmetic of base $p$, we define

$$[\![\Phi(\boldsymbol{x})]\!]_p := \left\{\boldsymbol{m} \in \mathbb{N}^d : \Phi[\boldsymbol{m}/\boldsymbol{x}] \text{ is valid}\right\},$$

[3] Other conventions are possible, e.g., asserting that $V_p(x, 0)$ holds if and only if $x = 1$ as in [3], but this does not change the sets of numbers definable in Büchi arithmetic.

where, for $\boldsymbol{m} = (m_1, \dots, m_d)$ and $\boldsymbol{x} = (x_1, \dots, x_d)$, $\Phi[\boldsymbol{m}/\boldsymbol{x}]$ is the formula obtained from replacing every $x_i$ by $m_i$ in $\Phi$. The set of sets of numbers definable in Presburger arithmetic is denoted by

$$\mathbf{PA} := \{[\![\Phi(x)]\!] : \Phi(x) \text{ is a formula of Presburger arithmetic}\}.$$

Analogously, we define the sets of numbers definable in fragments of Büchi arithmetic of base $p$ with a fixed number of quantifier-alternations as

$$\Sigma_i\text{-}\mathbf{BA}_p := \{[\![\Phi(x)]\!]_p : \Phi(x) \text{ is a } \Sigma_i\text{-formula of Büchi arithmetic of base } p\}.$$

Finally, $\mathbf{BA}_p := \bigcup_{i \geq 1} \Sigma_i\text{-}\mathbf{BA}_p$ denotes the sets of numbers definable in Büchi arithmetic of base $p$.

For separating existential Büchi arithmetic from full Büchi arithmetic, we employ some tools from enumerative combinatorics. As defined in [15], a formula of *parametric Presburger arithmetic* with parameter $t$ is a formula of Presburger arithmetic $\Phi_t$ in which atomic formulas are of the form $\boldsymbol{a} \cdot \boldsymbol{x} = c(t)$, where $c(t)$ is a univariate polynomial with indeterminate $t$ and coefficients in $\mathbb{Z}$. For $n \in \mathbb{N}$, we denote by $\Phi_n$ the formula of Presburger arithmetic obtained from replacing $c(t)$ in every atomic formula of $\Phi_t$ by the value of $c(n)$. We associate to a formula $\Phi_t(\boldsymbol{x})$ the counting function $\#\Phi_t(\boldsymbol{x})\colon \mathbb{N} \to \mathbb{N} \cup \{\infty\}$ such that

$$\#\Phi_t(\boldsymbol{x})(n) := \#[\![\Phi_n(\boldsymbol{x})]\!].$$

Throughout this paper, we constraint ourselves to formulas $\Phi_t(\boldsymbol{x})$ of parametric Presburger arithmetic in which $c(t)$ is the identity function and $\#\Phi_t(\boldsymbol{x})(n)$ is finite for all $n \in \mathbb{N}$.

**Definition 1.** *A function $f\colon \mathbb{N} \to \mathbb{Q}$ is an* eventual quasi-polynomial *if there exist a threshold $t \in \mathbb{N}$ and polynomials $p_0, \dots, p_{m-1} \in \mathbb{Q}[x]$ such that for all $n > t$, $f(n) = p_i(n)$ whenever $n \equiv i \bmod m$.*

Given an eventual quasi-polynomial $f$ with threshold $t$ and $n > t$, we denote by $f_n$ the polynomial $p_i$ such that $n \equiv i \bmod m$. We say that the polynomials $p_0, \dots, p_{m-1}$ *constitute* the eventual quasi-polynomial $f$. A result by Woods [15, Thm. 3.5(b)] shows that the counting functions associated to parametric Presburger formulas as defined above are eventual quasi-polynomial.

**Proposition 1 (Woods).** *Let $\Phi_t(\boldsymbol{x})$ be a formula of parametric Presburger arithmetic. Then $\#\Phi_t(\boldsymbol{x})$ is an eventual quasi-polynomial.*

*Semi-linear sets.* A result by Ginsburg and Spanier establishes that the sets of numbers definable in Presburger arithmetic are semi-linear sets [7]. A *linear set* in dimension $d$ is given by a base vector $\boldsymbol{b} \in \mathbb{N}^d$ and a finite set of period vectors $P = \{\boldsymbol{p}_1, \dots, \boldsymbol{p}_n\} \subseteq \mathbb{N}^d$ and defines the set

$$L(\boldsymbol{b}, P) := \{\boldsymbol{b} + \lambda_1 \cdot \boldsymbol{p}_1 + \cdots + \lambda_n \cdot \boldsymbol{p}_n : \lambda_i \in \mathbb{N}, 1 \leq i \leq n\}.$$

A *semi-linear set* is a finite union of linear sets. For a finite $B \subseteq \mathbb{N}^d$, we write $L(B, P)$ for $\bigcup_{\boldsymbol{b} \in B} L(\boldsymbol{b}, P)$. Semi-linear sets of the form $L(B, P)$ are called hybrid linear sets in [5], and it is known that the set of non-negative integer solutions of a system of linear Diophantine inequalities $S\colon \mathbf{A} \cdot \boldsymbol{x} \geq \boldsymbol{c}$ is a hybrid linear set [5].

Semi-linear sets in dimension one are also known as *ultimately periodic sets*. In this paper, we represent an ultimately periodic set as a four-tuple $U = (t, \ell, B, R)$, where $t \geq 0$ is a *threshold*, $\ell > 0$ is a *period*, $B \subseteq \{0, \ldots, t-1\}$ and $R \subseteq \{0, \ldots, \ell - 1\}$, and $U$ defines the set

$$[\![U]\!] := B \cup \{t + r + \ell \cdot i : r \in R, i \geq 0\}\,.$$

## 3 The inexpressiveness of existential Büchi arithmetic

We now establish the main result of this paper and show that the existential fragment of Büchi arithmetic is strictly less expressive than general Büchi arithmetic.

**Theorem 1.** *For any base $p \geq 2$, $\Sigma_1$-$\mathbf{BA}_p \neq \mathbf{BA}_p$. In particular, there exists a fixed regular language $L \subseteq \{0,1\}^*$ such that $[\![L]\!]_p \in \mathbf{BA}_p \setminus \Sigma_1$-$\mathbf{BA}_p$ for every base $p \geq 2$.*

Given a set $M \subseteq \mathbb{N}$, recall that for a fixed base $p \geq 2$, $d_M(n)$ counts the numbers of bit-length $n$ in base $p$ in $M$. As already discussed in the introduction, we prove Theorem 1 by characterizing the growth of $d_M$ for sets $M$ definable in Büchi arithmetic.

For any formula $\Phi(x)$ of existential Büchi arithmetic in prenex normal form, we can with no loss of generality assume that its matrix is in disjunctive normal form, i.e., a disjunction of *systems of linear Diophantine equations with valuation constraints*, each of the form

$$\mathbf{A} \cdot \boldsymbol{x} = \boldsymbol{c} \wedge \bigwedge_{i \in I} V_p(x_i, y_i),$$

where the $x_i$ and $y_i$ are unknowns from the vector of unknowns $\boldsymbol{x}$. For $M = [\![\Phi(x)]\!]_p$, in order to determine the growth of $d_M$, it suffices to determine the maximum growth occurring in any of its systems of linear Diophantine equations with valuation constraints in the matrix of $\Phi(x)$, which in turn can be obtained by analyzing the growth of the number of words accepted by a $p$-automaton defining the set of solutions of such a system.

Let $S\colon \mathbf{A} \cdot \boldsymbol{x} = \boldsymbol{c}$ be a system of linear Diophantine equations such that, throughout this section, $\mathbf{A}$ is an $m \times d$ integer matrix, and fix a base $p \geq 2$. Following Wolper and Boigelot [14], we define an automaton $A := (Q, \Sigma_p^d, \delta, \boldsymbol{q}_0, F)$ whose language encodes all solutions of $S$ over the alphabet $\Sigma_p$:

- $Q := \mathbb{Z}^m$,
- $\delta(\boldsymbol{q}, \boldsymbol{u}) := p \cdot \boldsymbol{q} + \mathbf{A} \cdot \boldsymbol{u}$ for all $\boldsymbol{q} \in Q$ and $\boldsymbol{u} \in \Sigma_p^d$,
- $\boldsymbol{q}_0 := \mathbf{0}$, and
- $F := \{\boldsymbol{c}\}$.

As discussed in [14], see also [8], only states $\boldsymbol{q}$ such that $\|\boldsymbol{q}\|_\infty \leq \|\mathbf{A}\|_{1,\infty}$ and $\|\boldsymbol{q}\|_\infty \leq \|\boldsymbol{c}\|_\infty$ can reach the accepting state. Hence, all words $w \in (\Sigma_p^d)^*$ such that $\mathbf{A} \cdot [\![w]\!] = \boldsymbol{c}$ only visit a finite number of states of $A$, and to obtain the $p$-automaton $A(S)$ defining the sets of solutions of $S$ we subsequently restrict $Q$ to only such states. The following lemma recalls an algebraic characterization of the reachability relation of $A(S)$ established in the proof of Proposition 14 in [8].

**Lemma 1.** *Let $\boldsymbol{q}, \boldsymbol{r} \in \mathbb{Z}^m$ be states of $A(S)$, $w \in (\Sigma_p^d)^n$ and $\boldsymbol{x} = [\![w]\!]_p$. Then $\boldsymbol{q} \xrightarrow{w} \boldsymbol{r}$ if and only if there is $y \in \mathbb{N}$ such that*

$$\boldsymbol{q} = \boldsymbol{r} \cdot y + \mathbf{A} \cdot \boldsymbol{x},\ \|\boldsymbol{x}\|_\infty < y,\ y = p^n.$$

Let $x$ be a distinguished variable of $\boldsymbol{x}$. For a word $w \in (\Sigma_p^d)^*$ encoding solutions of $S$, denote by $\pi_x(w)$ the word $v \in \Sigma_p^*$ obtained from projecting $w$ onto the component of $w$ corresponding to $x$. Let $q$ be a state of a $p$-automaton $A$, define the counting function $C_{q,x} \colon \mathbb{N} \to \mathbb{N}$ as

$$C_{q,x}(n) := \#\left\{\pi_x(w) : q \xrightarrow{w} q, w \in (\Sigma_p^d)^n\right\}.$$

We now show that for $p$-automata arising from systems of linear Diophantine equations, $C_{q,x}$ can be obtained from an eventual quasi-polynomial.

**Lemma 2.** *For the $p$-automaton $A(S)$ associated to $S\colon \mathbf{A} \cdot \boldsymbol{x} = \boldsymbol{c}$ with states $Q$ and all $q \in Q$, there is an eventual quasi-polynomial $f$ such that $C_{q,x}(n) = f(p^n)$ for all $n \in \mathbb{N}$. Moreover, for all sufficiently large $n \in \mathbb{N}$, $f_{p^n}$ is a linear polynomial.*

*Proof.* Let $q = \boldsymbol{q} \in \mathbb{Z}^d$. By Lemma 1, $\boldsymbol{q} \xrightarrow{w} \boldsymbol{q}$ for $w \in (\Sigma_p^d)^n$ if and only if there is a $y \in \mathbb{N}$ such that

$$\boldsymbol{q} = \boldsymbol{q} \cdot y + \mathbf{A} \cdot \boldsymbol{x},\ \|\boldsymbol{x}\|_\infty < y,\ y = p^n,$$

where $\boldsymbol{x} = [\![w]\!]_p$. The set of solutions of $S'\colon \mathbf{A} \cdot \boldsymbol{x} + \boldsymbol{q} \cdot y = \boldsymbol{q}, \|\boldsymbol{x}\|_\infty < y$ is a hybrid linear set $L(D, R) \subseteq \mathbb{N}^{d+1}$. Let $L(B, P) \subseteq \mathbb{N}^2$ be obtained from $L(D, R)$ by projecting onto the components corresponding to $x$ and $y$, and assume that $x$ corresponds to the first and $y$ to the second component of $L(B, P)$. Let $M_t := \mathbb{N} \times \{t\}$ and

$$f(t) := \#(L(B, P) \cap M_t).$$

Observe that $C_{q,x}(n) = f(p^n)$ and that $f(n)$ is finite for all $n \in \mathbb{N}$ due to the constraint $x < y$. Let $P = \{\boldsymbol{p}_1, \ldots, \boldsymbol{p}_k\}$, the following formula of parametric Presburger arithmetic defines $L(B, P) \cap M_t$:

$$\Phi_t(x, y) := \exists z_1 \cdots \exists z_k \colon \bigvee_{\boldsymbol{b} \in B} \begin{pmatrix} x \\ y \end{pmatrix} = \boldsymbol{b} + \sum_{i=1}^{k} \boldsymbol{p}_i \cdot z_i \wedge y = t$$

Thus, $f = \#\Phi_t(x, y)$ and, by application of Proposition 1, $f$ is an eventual quasi-polynomial.

Since $C_{q,x}(n) \leq p^n - 1$ for all $n \in \mathbb{N}$, we in particular have that all polynomials $f_{p^n}$ constituting $f$ are linear as they would otherwise outgrow $C_{q,x}$. □

The next step is to lift Lemma 2 to systems of linear Diophantine equations with valuation constraints. To this end, we define a DFA whose language encodes the set of all solutions of predicates of the form $V_p(x, y)$. Formally, for $S\colon V_p(x, y)$ we define $A(S) := (Q, \Sigma_p^d, \delta, q_0, F)$ such that

- $Q := \{0, 1\}$,
- $\delta(0, \boldsymbol{u}) := 0$ for all $\boldsymbol{u} \in \Sigma_p^d$ such that $\pi_x(\boldsymbol{u}) = 0$,
- $\delta(0, \boldsymbol{u}) := 1$ for all $\boldsymbol{u} \in \Sigma_p^d$ such that $\pi_x(\boldsymbol{u}) = 1$ and $\pi_y(\boldsymbol{u}) > 0$,
- $\delta(1, \boldsymbol{u}) := 1$ for all $\boldsymbol{u} \in \Sigma_p^d$ such that $\pi_x(\boldsymbol{u}) = \pi_y(\boldsymbol{u}) = 0$,
- $q_0 := 0$, and
- $F := \{1\}$.

For $S\colon \mathbf{A} \cdot \boldsymbol{x} = \boldsymbol{c} \wedge \bigwedge_{1 \le i \le \ell} V_p(x_i, y_i)$, we denote by $A(S)$ the DFA that can be obtained from the standard product construction on all DFA for the atomic formulas of $S$. Hence, the set of states of $A(S)$ is a finite subset of $\mathbb{Z}^m \times \{0, 1\}^\ell$. We now show that the number of words along a cycle of $A(S)$ can also be obtained from an eventual quasi-polynomial.

**Lemma 3.** *Let $S$ be a system of linear Diophantine equations with valuation constraints with the associated DFA $A(S)$ with states $Q$, and let $q \in Q$. There is an eventual quasi-polynomial $f$ such that $C_{q,x}(n) = f(p^n)$. Moreover, $f_{p^n}$ is a linear polynomial for all $n \in \mathbb{N}$.*

*Proof.* Let $S\colon \mathbf{A} \cdot \boldsymbol{x} = \boldsymbol{c} \wedge \bigwedge_{1 \le i \le \ell} V_p(x_i, y_i)$, we have $Q \subseteq \mathbb{Z}^m \times \{0, 1\}^\ell$ and thus $q = (\boldsymbol{q}, b_1, \ldots, b_\ell) \in Q$. Any self-loop $q \xrightarrow{w}_S q$ with $q = (\boldsymbol{q}, b_1, \ldots, b_\ell)$ is a self-loop for the DFA induced by the system of linear Diophantine equations $\mathbf{A} \cdot \boldsymbol{x} = \boldsymbol{c}$ with the additional requirement that $\pi_{x_i}([\![w]\!]_p) = 0$ for all $1 \le i \le \ell$ and furthermore $\pi_{y_i}([\![w]\!]_p) = 0$ whenever $b_i = 1$. Thus $(\boldsymbol{q}, \boldsymbol{0}) \xrightarrow{w}_{S'} (\boldsymbol{q}, \boldsymbol{0})$ where

$$S'\colon \mathbf{A} \cdot \boldsymbol{x} = \boldsymbol{c} \wedge \bigwedge_{1 \le i \le \ell} x_i = 0 \wedge \bigwedge_{1 \le i \le \ell, b_i = 1} y_i = 0\,.$$

Conversely, $(\boldsymbol{q}, \boldsymbol{0}) \xrightarrow{w}_{S'} (\boldsymbol{q}, \boldsymbol{0})$ immediately gives $q \xrightarrow{w}_S q$. The statement is now an immediate consequence of the application of Lemma 2 to $S'$. □

We will from now on implicitly apply Lemma 3. As a first application, we show that Lemma 3 allows us to classify the DFA associated to a system of linear Diophantine equations with valuation constraints.

**Lemma 4.** *The DFA $A(S)$ associated to a system of linear Diophantine equations with valuation constraints $S$ with states $Q$ has either of the following properties:*

*(i) there is $q \in Q$ such that $C_{q,x}$ is an eventual quasi-polynomial $f$ and $f_{p^n}$ is a non-constant polynomial for infinitely many $n \in \mathbb{N}$; or*
*(ii) there is a constant $d \ge 0$ such that $C_{q,x}(n) \le d$ for all $q \in Q$ and $n \in \mathbb{N}$.*

*Proof.* Suppose $A(S)$ has Property (i). For a contradiction, suppose $d \geq 0$ exists. Let $f$ be the eventual quasi-polynomial from Property (i). Every non-constant polynomial $f_{p^n}$ constituting $f$ is of the form $a \cdot x + b$ with $a > 0$. As there are infinitely many such $n$, there is some linear polynomial $g(x) = a \cdot x + b$ such that $g = f_{p^n}$ for infinitely many $n \in \mathbb{N}$. Hence $g(p^n) > d$ for some sufficiently large $n \in \mathbb{N}$.

For the converse, suppose that $A(S)$ does not have Property (i). Then there are $\ell, m > 0$ such that all $f_{p^n}$ are constant polynomials bounded by some value $m \in \mathbb{N}$ for all $n \geq \ell$, $q \in Q$ and $f = C_{q,x}$. Hence we can choose $d = \max(\{C_{q,x}(n) : q \in Q, 0 < n \leq \ell\} \cup \{m\})$. □

We are now in a position to prove a dichotomy of the growth of the number of words accepted by a DFA corresponding to a system of linear Diophantine equations with valuation constraints.

**Lemma 5.** *Let $S$ be a fixed system of linear Diophantine equations with valuation constraints with the associated DFA $A(S)$. Let $L = \pi_x(L(A(S)))$, then either*

*(i) $d_L(n) \geq c \cdot p^n$ for some fixed constant $c > 0$ and infinitely many $n \in \mathbb{N}$; or*
*(ii) $d_L(n) = O(n^c)$ for some fixed constant $c \geq 0$.*

*Proof.* Let $A(S)$ have the set of states $Q$, initial state $q_0$ and final state $q_f$. The DFA $A(S)$ has one of the two properties stated in Lemma 4.

If $A(S)$ has the Property (i) of Lemma 4 then consider $q \in Q$ such that $C_{q,x}$ is an eventual quasi-polynomial $f$ such that $f_{p^n}$ is non-constant for infinitely many $n \in \mathbb{N}$, and let $i_1 < i_2 < \ldots \in \mathbb{N}$ be such that all $f_{p^{i_j}}$ are the same non-constant polynomial $a \cdot x + b$. Consider $v$ and $w$ such that $q_0 \xrightarrow{v} q \xrightarrow{w} q_f$. Then for all sufficiently large $j$ we have

$$d_L(i_j + |v| + |w|) \geq a \cdot p^{i_j} + b \geq c \cdot p^{(i_j+|v|+|w|)}$$

for some fixed constant $c > 0$.

Otherwise, $A(S)$ has the Property (ii) of Lemma 4, and there is some fixed $d \geq 0$ such that $C_{q,x}(n) \leq d$ for all $n \in \mathbb{N}$ and $q \in Q$. Every $w \in L$ such that $|w| = n$ can uniquely be decomposed as $w = v_0 w_1 v_1 w_2 \cdots w_k v_k$ for some $k \leq |Q|$ such that

$$q_0 \xrightarrow{v_0} q_{a_1} \xrightarrow{w_1} q_{a_1} \xrightarrow{v_1} q_{a_2} \xrightarrow{w_2} q_{a_2} \xrightarrow{v_2} q_{a_3} \cdots \xrightarrow{w_k} q_{a_k} \xrightarrow{v_k} q_{a_{k+1}}, \tag{1}$$

where $q_{a_{k+1}} = q_f$, $q_{a_i} \neq q_{a_j}$ for all $i \neq j$ and each $q_{a_i} \xrightarrow{v_i} q_{a_{i+1}}$ corresponds to a loop-free path in $A(S)$. Since $C_{q,x} \leq d$, there are at most $d^k \leq d^{(\#Q)}$ words $u \in L$ of length $n$ that have the same sequence of states in the decomposition of Eq. (1) at the same position where they occur in $w$. Moreover, there are at most $\binom{n}{2k} \leq \binom{n}{2 \cdot \#Q} \leq n^{(2 \cdot \#Q)}$ possibilities at which the states $q_{a_i}$ can appear in any $u \in L$ of length $n$ for any particular sequence of states in the decomposition of Eq. (1). Finally, there are at most $(\#Q)^{(\#Q)}$ such sequences. We thus derive

$$d_L(n) \leq (\#Q)^{\#Q} \cdot n^{(2 \cdot \#Q)} \cdot d^{(\#Q)} = O(n^c)$$

for some constant $c \geq 0$. □

**Corollary 1.** *Let $\Phi(x)$ be a fixed formula of existential Büchi arithmetic of base $p \geq 2$. Let $M = [\![\Phi(x)]\!]_p$, then either:*

*(i) $d_M(n) \geq c \cdot p^n$ for some fixed constant $c > 0$ and infinitely many $n \in \mathbb{N}$; or*
*(ii) $d_M(n) = O(n^c)$ for some fixed constant $c \geq 0$.*

*Proof.* Without loss of generality we may assume that $\Phi(x)$ is in disjunctive normal form such that $\Phi(x) = \bigvee_{i \in I} \Phi_i(x)$ and each $\Phi_i(x)$ is a system of linear Diophantine equations with valuation constraints $S_i$. For $M_i = [\![\Phi_i(x)]\!]_p$, we obtain $d_{M_i}$ by application of Lemma 5. If there is a constant $c \geq 0$ such that $d_{M_i} = O(n^c)$ for all $i \in I$ then $d_M = O(n^c)$. Otherwise, if there is some $i \in I$ such that $d_{M_i}(n) \geq c \cdot p^n$ for some constant $c > 0$ and infinitely many $n \in \mathbb{N}$ then $d_M(n) \geq c \cdot p^n$ for infinitely many $n \in \mathbb{N}$. □

As an immediate consequence of Corollary 1, we obtain:

**Corollary 2.** *Let $p \geq 2$ and $M \subseteq \mathbb{N}$ such that $f = o(d_M)$ for any $f = O(n^c)$, $c \geq 0$, and $d_M = o(p^n)$. Then $M \notin \Sigma_1$-$\mathbf{BA}_p$.*

For any $p \geq 2$, consider $L = \{01, 10\}^* \subseteq \Sigma_p^*$ and $M = [\![L]\!]_p$. We have $d_M(n) = \Theta(2^{n/2})$, and thus Corollary 2 yields $M \notin \Sigma_1$-$\mathbf{BA}_p$. However, since $M$ is $p$-regular, we have $M \in \mathbf{BA}_p$. This concludes the proof of Theorem 1.

## 4 Expressive completeness of the $\Sigma_2$-fragment of Büchi arithmetic

For a regular language $L \subseteq (\Sigma_p^d)^*$ given by a DFA, Villemaire shows in the proof of Theorem 2.2 in [13] how to construct a $\Sigma_3$-formula of Büchi arithmetic $\Phi_L(\boldsymbol{x})$ such that $[\![\Phi_L(\boldsymbol{x})]\!]_p = [\![L]\!]_p$. This construction is modularized and relies on an existential formula $\Phi_{p,j}(x, y)$ expressing that *"x is a power of p and the coefficient of this power of p in the representation of y in base p is j"*:

$$\Phi_{p,j}(x, y) \equiv P_p(x) \wedge \exists t\, \exists u\, \exists z \colon \big(y = z + j \cdot x + t\big) \wedge (z < x) \wedge \wedge ((V_p(u, t) \wedge x < u) \vee t = 0)\,.$$

The only reason why $\Phi_L(\boldsymbol{x})$ in [13] is a $\Sigma_3$-formula is that $\Phi_{p,j}(x, y)$ appears in an implication both as antecedent and as consequent inside an existential formula. Thus, if one could additionally define $\Phi_{p,j}(x, y)$ by a $\Pi_1$-formula then $\Phi_L(\boldsymbol{x})$ immediately becomes a $\Sigma_2$-formula. That is, however, not difficult to achieve by defining:

$$\widetilde{\Phi}_{p,j}(x, y) := P_p(x) \wedge \forall s\, \forall t\, \forall u\, \forall z \colon$$
$$\Big(\neg(s = z + j \cdot x + t) \vee (z \geq x) \vee (\neg V_p(u, t) \vee x \geq u) \wedge \neg(t = 0))\Big) \to \neg(s = y)\,.$$

Note that the order relation can also be expressed by a universal formula: $x \leq y$ if and only if $\forall z \colon (y + z = x) \to (z = 0)$. Thus, $\widetilde{\Phi}_{p,j}(x, y)$ is indeed a $\Pi_1$ formula.

Combining $\widetilde{\Phi}_{p,j}(x, y)$ with the results in [13], we obtain that the $\Sigma_2$-fragment of Büchi arithmetic is expressively complete.

**Theorem 2.** *For any base $p \geq 2$, $\Sigma_2$-$\mathbf{BA}_p = \mathbf{BA}_p$.*

## 5 Existential Büchi arithmetic defines regular languages of polynomial growth

For a language $L \subseteq \Sigma^*$, Szilard et al. [11] say that $L$ has *polynomial growth* if $d_L(n) = O(n^c)$ for some constant $c \geq 0$ and all $n \in \mathbb{N}$. One of the main results of [11] is that a regular language $L$ has polynomial growth if and only if $L$ can be represented as a finite union of regular expressions of the form

$$v_0 w_1^* v_1 \cdots v_{k-1} w_k^* v_k\,. \tag{2}$$

Denote by

$$\mathbf{PREG}_p := \big\{ [\![L]\!]_p : L \subseteq \Sigma_p^*,\ L \text{ is a regular language of polynomial growth} \big\}$$

the numerical encoding of all regular languages of polynomial growth in base $p$. We show in this section that existential Büchi arithmetic defines any regular language of the form in Eq. (2). This immediately gives the following theorem.

**Theorem 3.** *For any base $p \geq 2$, $\mathbf{PREG}_p \subseteq \Sigma_1\text{-}\mathbf{BA}_p$.*

We first require a couple of abbreviations. Define

$$W_p(x, y) := P_p(y) \wedge x < y \leq p \cdot x,$$

which expresses that $y$ is the smallest power of $p$ strictly greater than $x$.

Let $\ell > 0$, Lohrey and Zetzsche introduce in [9] the predicate $S_\ell(x, y)$ which holds whenever

$$x = p^r \text{ and } y = p^{r+\ell\cdot i} \text{ for some } i, r \geq 0\,.$$

They show that $S_\ell(x, y)$ is definable in existential Büchi arithmetic. Since $y = p^{\ell \cdot i} \cdot x$ if and only if $y \equiv x \bmod (p^\ell - 1)$, one can obtain $S_\ell$ as

$$S_\ell(x, y) := P_p(x) \wedge P_p(y) \wedge \exists z \colon (y - x = (p^\ell - 1) \cdot z) \wedge y \geq x\,.$$

We slightly generalize $S_\ell$. Let $U \subseteq \mathbb{N}$, define the predicate $S_U(x, y)$ to hold whenever

$$x = p^r \text{ and } y = p^{r+u} \text{ for some } r \geq 0 \text{ and } u \in U\,.$$

**Lemma 6.** *For any ultimately periodic set $U \subseteq \mathbb{N}$, the predicate $S_U(x, y)$ is definable in existential Büchi arithmetic*

*Proof.* Suppose that $U$ is given as $(t, \ell, B, R)$, we define

$$S_U(x, y) := P_p(x) \wedge P_p(y) \wedge \bigvee_{b \in B} y = p^b \cdot x \vee \bigvee_{r \in R} S_\ell(p^{t+r} \cdot x, y)\,.$$

□

Towards proving Theorem 3, we now show that we can define $[\![w^*]\!]_p$ for any $w \in \Sigma_p$.

**Lemma 7.** *For any $w \in \Sigma_p^*$, $[\![w^*]\!]_p$ is definable by a formula of existential Büchi arithmetic $\Phi_{w^*}(x)$.*

*Proof.* Let $m = p^\ell$ be the smallest power of $p$ greater than $[\![w]\!]_p$. Then for any $k > 0$,

$$[\![w^k]\!]_p = [\![w]\!]_p \cdot \sum_{i=0}^{k-1} m^i = [\![w]\!]_p \cdot \frac{m^k - 1}{m - 1}\,.$$

It follows that $[\![w^*]\!]_p$ is defined by

$$\Phi_{w^*}(x) := x = 0 \vee \exists y\colon S_\ell(m, y) \wedge (m-1) \cdot x = [\![w]\!]_p \cdot (y-1)\,.$$

□

Building upon Lemma 7, we now show that, for any $w \in \Sigma_p$, we can define $[\![w^+]\!]_p$ shifted to the left by a number of zeros specified by an ultimately periodic set.

**Lemma 8.** *Let $w \in \Sigma_p^*$ and $U$ be an ultimately periodic set. Then $[\![w^+ 0^U]\!]_p$ is definable by a formula of existential Büchi arithmetic $\Phi_{U,w^+}(x)$.*

*Proof.* The case $w \in 0^*$ is trivial. Thus, let $w = w' \cdot w_0$ such that $w' \in \Sigma_p^* \cdot (\Sigma_p \setminus \{0\})$ and $w_0 \in 0^*$. Observe that for $i < j$, $[\![w^j]\!]_p - [\![w^i]\!]_p = [\![w^{j-i}0^i]\!]_p$. We define

$$\Phi_{U,w^+}(x) := \exists y\, \exists z\colon y < z \wedge \Phi_{w^*}(y) \wedge \Phi_{w^*}(z) \wedge \bigvee_{0 \le i < |w|} x = p^i \cdot (z - y) \wedge$$
$$\wedge\, \exists s\, \exists t\colon S_U(1, s) \wedge V_p(t, x) \wedge t = p^{|w_0|+1} \cdot s\,.$$

The first line defines the set $[\![w^+ 0^*]\!]_p$, whereas the second line ensures that the tailing number of zeros is in the set $U + |w_0|$. □

We have now all the ingredients to prove the following key proposition.

**Proposition 2.** *Let $L = v_0 w_1^* v_1 \cdots v_{k-1} w_k^* v_k$. Then $[\![L]\!]_p$ is definable in existential Büchi arithmetic.*

*Proof.* The proposition follows from showing the statement for languages of the form

$$L' = v_0 w_1^+ v_1 \cdots v_{k-1} w_k^+ v_k\,.$$

We show the statement by induction on $k$. The induction base case $k = 0$ is trivial. For the induction step, assume that for $M = v_1 w_2^+ v_2 \cdots v_{k-1} w_k^+ v_k$, $[\![M]\!]_p$ is defined by a formula $\Phi_k(x)$ of existential Büchi arithmetic, and let $v_0, w_1 \in \Sigma_p^*$.

We first show how to define $N = w_1^+ v_1 w_2^+ v_2 \cdots v_{k-1} w_k^+ v_k$. To this end, factor $M = M_0 \cdot M'$, where $M_0 \subseteq 0^*$ and $M \subseteq (\Sigma_p \setminus \{0\}) \cdot \Sigma_p^*$. Observe that $[\![M']\!]_p = [\![\Phi_k(x)]\!]_p$, and that both $U = \{|w| : w \in M\}$ and $V = \{|w| : w \in M_0\}$ are ultimately periodic sets, cf. [6,12]. We moreover assume that $w_1 \notin 0^*$, otherwise we are done. Factor $w_1 = w' \cdot w_0$ such that $w' \in \Sigma_p^* \cdot (\Sigma_p \setminus \{0\})$ and $w_0 \in 0^*$.

Recall that $W_p(x,y)$ holds if and only if $y$ is the smallest power of $p$ strictly greater than $x$, and define

$$\Psi_{k+1}(x) := \exists y\, \exists z\colon \Phi_k(y) \wedge \Phi_{U,w^+}(z) \wedge x = y + z \wedge \\ \wedge \exists s\, \exists t\colon W_p(y,s) \wedge S_V(s,t) \wedge V_p(p^{|w_0|+1} \cdot t, z)\,.$$

The first line composes $x$ as the sum of some $y \in [\![M]\!]_p$ and $z \in [\![w^+0^U]\!]_p$. The second line ensures that the number of zeros between the leading bit of $y$ and the last non-zero digit of $z$ in their $p$-ary expansion is in $V + |w_0|$. Thus, $[\![N]\!]_p = [\![\Psi_{k+1}(x)]\!]$.

We now show how to define $L'$ along similar lines. To this end, factor $N = N_0 \cdot N'$ such that $N_0 \subseteq 0^*$ and $N' \subseteq (\Sigma_p \setminus \{0\}) \cdot \Sigma_p^*$, and let $T = \{|w| : w \in N_0\}$, which is an ultimately periodic set. We now obtain the desired formula of existential Büchi arithmetic as

$$\Phi_{k+1}(x) := \exists y\, \exists z\colon x = y + p \cdot z \cdot [\![v_0]\!]_p \wedge \Psi_{k+1}(y) \wedge \exists s\colon W_p(y,s) \wedge S_T(s,z)\,.$$

□

Since we can define any regular language of the form (2) in existential Büchi arithmetic via Proposition 2, we can define a finite union of such languages and thus define all regular languages of polynomial growth in existential Büchi arithmetic. This completes the proof of Theorem 3.

Note that $\mathbf{PREG}_p \not\subseteq \mathbf{PA}$ for any base $p \geq 2$: since $M = [\![\Phi(x)]\!]$ is ultimately periodic for any formula $\Phi(x)$ of Presburger arithmetic, whenever $[\![\Phi(x)]\!]$ is infinite it follows that $d_M(n) = \Omega(p^n)$, i.e., not of polynomial growth.

## 6 Conclusion

The main result of this paper is that existential Büchi arithmetic is strictly less expressive than full Büchi arithmetic of any base. This is in contrast to Presburger arithmetic, for which it is known that its existential fragment is expressively complete.

When considered as the first-order theory of the structure $\langle \mathbb{N}, 0, 1, + \rangle$, Presburger arithmetic does not have a quantifier elimination procedure. The extended structure $\langle \mathbb{N}, 0, 1, +, \{c|\cdot\}_{c>1} \rangle$, however, admits quantifier elimination. Those additional divisibility predicates are definable in existential Presburger arithmetic. Our main result shows that even if we extended the structure underlying Büchi arithmetic with predicates definable in existential Büchi arithmetic, the resulting first-order theory would not admit quantifier-elimination. On the positive side, Benedikt et al. [1, Thm. 3.1] give an extension of Büchi arithmetic which has quantifier elimination.

We conclude this paper with an interesting yet likely challenging open problem: Is it decidable whether a set definable in Büchi arithmetic is definable in existential Büchi arithmetic?

**Acknowledgments.** We would like to thank Dmitry Chistikov and Alex Fung for inspiring discussions on the topics of this paper, and the FoSSaCS'21 reviewers for their comments and suggestions.

This work is part of a project that has received funding from the European Research Council (ERC) under the European Union's Horizon 2020 research and innovation programme (Grant agreement No. 852769, ARiAT).
